# Medical Men in Politics

**Published:** 1896-01

**Authors:** 


**Medical Men in Politics.**—The *Boston Medical and Surgical Journal* comments upon the slender influence over wholesome legislation that comes within the power of the profession. Doubtless every community has its quota of physicians who are not too much occupied with professional work to give a share of their time to political interests. Doubtless, also, a small proportion is willing to engage in legislative duty if called upon so to do by the community wherein they respectively dwell. But it is unfortunately, also, the case that those who "go to the front," from our profession, for such legislative functions are not from the representative men in medicine; they are ordinarily chosen to represent something bearing upon "spoils" or "bone-hunting." Their interest with the sanitary and medical interests of their constituents are commonly subordinated to relatively small issues. Then, as the *Journal* remarks, the kind of work that is called "political" is, for the most part, distateful to men of the type who make a success in medical life, and may even be by them regarded as incompatible with professional success. The *Journal* closes its article by citing an instance which speaks for itself, regarding the kind of selection of a politico-medical representative, that may sometimes be made even in enlightened New England:

"A conspicuous example of the influence of the medical man in politics in retarding legislation for the benefit of the public health, was the attitude taken by Senator Gallinger, of New Hampshire, who had been a medical practitioner in that State, on the bill appropriating money for a bacteriologic laboratory and disinfecting service and for the distribution of antitoxin in the District of Columbia. It will be remembered that his attitude, as noted in our issue of Feb. 7, 1895, was against the passage of the bill, and that his influence was thrown squarely against this legislation for the public health rather than in favor of it. There can be no doubt that there is need of the representation of the medical profession in our legislatures by the right class of men from the highest ranks of the profession. And inasmuch as active participation in legislative work is impossible for the rank and file of the profession, it becomes the duty and privilege of what may be called the medical leisure class to do this work; we mean those of independent means, who are not dependent upon the practice of their profession for their living."

Senator Gallinger is supposed to be a homeopath and his actions must be viewed from that standpoint.—*N. Y. Med. Record.*

We, in Texas, have had similar experiences; the homeopaths have defeated every attempt on the part of the State Medical Association to procure medical legislation to prevent quackery, —perhaps conscious, themselves, of their own demerits, and that many of their class are arrant quacks. The ring-leaders in the opposition were so narrow-minded and ignorant that actually they protested against "State medicine," under the impression that it meant State control of practice,—had no conception of that grand idea of utilizing medical knowledge in the prevention of disease. State medicine and preventive medicine are the same, and embrace measures looking to the protection of unborn generations as well as those now living; for instance, castration, prohibition of matrimony among certain persons, in the interest of society; the suppression of the liquor traffic; prohibition—are all measures which come properly within the purview of State medicine, as well as vaccination and quarantine, and yet *Fisher*, —the loud-mouthed and shallow disciple of Hahneman—the worst Hahne*maniac* of the lot—cried out against State medicine as "class legislation." Can such things be? In the language of Bret Harte, "We are ruined by homeopathic cheap labor."

